# Phase angle from bioelectrical impedance analysis is a useful indicator of muscle quality

**DOI:** 10.1002/jcsm.12860

**Published:** 2021-11-30

**Authors:** Yasunori Akamatsu, Toru Kusakabe, Hiroshi Arai, Yuji Yamamoto, Kazuwa Nakao, Kentaro Ikeue, Yuki Ishihara, Tetsuya Tagami, Akihiro Yasoda, Kojiro Ishii, Noriko Satoh‐Asahara

**Affiliations:** ^1^ Department of Endocrinology, Metabolism, and Hypertension Research, Clinical Research Institute National Hospital Organization Kyoto Medical Center Kyoto Japan; ^2^ Health Administration Center Kyoto Institute of Technology Kyoto Japan; ^3^ Health and Medical Services Center Shiga University Hikone Japan; ^4^ Medical Innovation Center Kyoto University Graduate School of Medicine Kyoto Japan; ^5^ Graduate School of Health and Sports Science Doshisha University Kyotanabe Japan; ^6^ Department of Endocrinology and Metabolism National Hospital Organization Kyoto Medical Center Kyoto Japan; ^7^ Clinical Research Institute National Hospital Organization Kyoto Medical Center Kyoto Japan; ^8^ Faculty of Health and Sports Science Doshisha University Kyotanabe Japan

**Keywords:** Phase angle, Sarcopenia, Muscle quality, Body composition, Bioelectrical impedance

## Abstract

**Background:**

Methods that facilitate muscle quality measurement may improve the diagnosis of sarcopenia. Current research has focused on the phase angle (PhA) obtained through bioelectrical impedance analysis (BIA) as an indicator of cellular health, particularly cell membrane integrity and cell function. The current study therefore aimed to evaluate the relationship between the PhA and muscle quality and muscle‐related parameters and to determine factors associated with the PhA. Moreover, we attempted to determine the cut‐off value of PhA for predicting sarcopenia.

**Methods:**

First‐year university students (830 male students, 18.5 ± 0.6 years old; 422 female students, 18.3 ± 0.5 years old) and community‐dwelling elderly individuals (70 male individuals, 74.4 ± 5.5 years old; 97 female individuals, 73.1 ± 6.4 years old) were included. PhA and other body composition data were measured using BIA, while muscle quality was calculated by dividing handgrip strength by upper limbs muscle mass. The relationship between PhA and the aforementioned parameters were then analysed, after which the cut‐off value of PhA for predicting sarcopenia was examined.

**Results:**

Multiple linear regression analysis revealed that age, skeletal muscle mass index (SMI), and muscle quality were independently associated with PhA in both sexes [male (age: standardized regression coefficient (*β*) = −0.43, *P* < 0.001, SMI: *β* = 0.61, *P* < 0.001, muscle quality: *β* = 0.13, *P* < 0.001) and female (age: *β* = −0.56, *P* < 0.001, SMI: *β* = 0.52, *P* < 0.001, muscle quality: *β* = 0.09, *P* = 0.007)]. Participants with sarcopenia had a significantly lower PhA compared with those without it (sarcopenia vs. non‐sarcopenia: young male participants, 5.51 ± 0.41° vs. 6.25 ± 0.50°, *P* < 0.001; young female participants, 4.88 ± 0.16° vs. 5.37 ± 0.44°, *P* = 0.005; elderly female participants: 4.14 ± 0.29° vs. 4.63 ± 0.42°, *P* = 0.009). Although no significant findings were observed in elderly male participants, the same tendency was noted. Receiver operating characteristic (ROC) curve analysis indicated that PhA had good predictive ability for sarcopenia in young male, elderly male, young female, and elderly female participants (area under the ROC curve of 0.882, 0.838, 0.865, and 0.850, with cut‐off PhA values of 5.95°, 5.04°, 5.02°, and 4.20° for predicting sarcopenia, respectively).

**Conclusions:**

The PhA reflected muscle quality and exhibited good accuracy in detecting sarcopenia, suggesting its utility as an index for easily measuring muscle quality, which could improve the diagnosis of sarcopenia.

## Introduction

Reports have shown that the prevalence of sarcopenia can range from 5% to 13% among individuals between the age of 60 and 70 and from 11% to 50% among elderly individuals over 80 years.^1^ According to a recent systematic review and meta‐analysis, the prevalence of sarcopenia in the world is 10% [95% confidence interval (CI) = 8–12%] in male participants and 10% (95% CI = 8–13%) in female participants, respectively.^2^ The prevention and improvement of sarcopenia, which remains a serious problem worldwide, has become an important issue. Sarcopenia is mainly diagnosed based on guidelines proposed by the European Working Group on Sarcopenia in Older People (EWGSOP) in 2010^3^ and subsequently revised in 2018 (EWGSOP2).^4^ Essentially, sarcopenia has been diagnosed based on whether ‘muscle strength’, ‘muscle quantity’, and ‘physical performance’ satisfy certain criteria.

Although muscle mass remains the primary factor of muscle strength, studies have shown that muscle strength is only moderately correlated with muscle cross‐sectional area and muscle thickness among living bodies.^5^, ^6^ Furthermore, both muscle mass and strength decline along with age; however, the decline in muscle strength is substantially greater than the decline in muscle mass.^7^, ^8^ Thus, a decrease in muscle mass alone cannot fully explain the loss of muscle strength.^9^

The skeletal myocyte gap contains not only muscle fibres (muscle cells) but also fibrous tissue, intramuscular extracellular fat, and extracellular fluid. During muscle atrophy, qualitative changes, such as an increase in intramuscular fat and fibrous tissue, occur in addition to quantitative changes (e.g. a decrease in muscle cross‐sectional area),^10^ thereby suggesting the importance of assessing muscle quality. Given that the effects of muscle quality deterioration have also been considered when diagnosing sarcopenia, guidelines have indicated the need for examining muscle quality.^4^ Accordingly, the EWGSOP2 guideline has defined ‘muscle quality’ as that ‘referring both to micro‐ and macroscopic changes in muscle architecture and composition, and to muscle function delivered per unit of muscle mass’.^4^ Nonetheless, no clear criteria have been established for diagnosing sarcopenia.

Highly sensitive imaging modalities, such as computed tomography^11^ and magnetic resonance imaging,^12^ have been used to assess muscle quality by measuring fat infiltration into the muscle and evaluating muscle attenuation. Moreover, muscle biopsy has been used to directly examine the muscle architecture and composition.^13^ However, although these methods can provide precise measurements, obtaining such measurements has remained challenging given the need for invasive procedures, large‐scale equipment, prolonged restraint, and skilled technicians. Although echo intensity obtained from ultrasonography images has also been used to assess muscle quality,^14^ reproducibility of results has remained a concern given the differences in measurement techniques between each examiner.

Alternatively, the ratios of muscle strength to appendicular skeletal muscle mass (ASM)^15^ or muscle volume^16^ have also been used as indicators of muscle quality. Despite being simple ratios, they have attracted much attention given studies associating with cardiovascular diseases.^17^ Nonetheless, given the lack of a universal consensus thus far, methods that can easily assess muscle quality in clinical practice have been desired.

Interestingly, bioelectrical impedance analysis (BIA) has become quite a popular method for estimating body composition, including muscle mass, considering that it is non‐invasive, inexpensive, portable, and easy and quick to use.^18^ To determine body composition, BIA measures the human body's impedance (Z), which is the electrical opposition to the alternating current (AC) of the body composed of resistance (R) and reactance (Xc) represented by the following formula: Z^2^ = R^2^ + Xc^2^.^19^ After substituting the obtained impedance values and participant height into the regression equations for each studied population, BIA can estimate lean body mass and body water content, among others.

The phase angle (PhA), which is calculated using the arctangent value of the ratio of Xc to R, is independent of conventional regression equations for estimating body composition.^20^ When an AC flows through the human body, healthy cell membranes function as capacitors that store electrical energy, consequently causing a delay in its flow. This lag in the current that penetrates cell membranes and tissue interfaces creates the phase difference between the current and voltage, which is expressed as the PhA.^21^

Recent studies have focused on the PhA as an indicator of cellular health, particularly reflecting cell membrane integrity and cell function.^22^ Accordingly, the PhA has been reported to be associated with age,^23^, ^24^, ^25^ sex,^23^, ^24^, ^25^ and nutrition.^26^ Currently, studies have reported low PhA values among patients with HIV^27^, ^28^ and cancer,^29^, ^30^, ^31^ as well as those on haemodialysis,^32^ with PhA being used in their treatment. Moreover, one study reported that lower PhA was correlated with poor muscle function, particularly low muscle volume or strength.^33^

However, no study has determined whether muscle quality and quantity are associated with PhA. As such, the present study aimed to evaluate the association between PhA and muscle parameters and determine factors associated with PhA. Moreover, we attempted to determine cut‐off values of PhA for detecting sarcopenia. Granting that muscle quality is associated with PhA, we believe that PhA could be a useful and convenient index of muscle quality, which can help improve the diagnosis of sarcopenia.

## Methods

### Participants

Participants were recruited during health check‐ups held by the town or universities. Young participants included first‐year university students of Kyoto Institute of Technology or Shiga University aged 18 to 20, while the elderly participants comprised community‐dwelling elderly individuals residing in Seika town, Kyoto.

The exclusion criteria were as follows: (i) unable to maintain a standing position, (ii) presence of cardiac pacemakers, (iii) presence of artificial joints, (iv) apparent oedematous, (v) pregnant or suspected of being pregnant, and (vi) considered unsuitable for study participation.

Consent to participate was obtained from all participants before study inclusion. This study was reviewed and approved by the Ethics Committee of National Hospital Organization Kyoto Medical Center (approval number: 18‐106 in young participants and 18‐107 in elderly participants) and was conducted in compliance with the ethical principles stated in the Declaration of Helsinki.

### Data collection

#### Bioelectrical impedance and phase angle

A multi‐frequency segmental body composition analyser (MC‐780A‐N, TANITA Co., Ltd., Tokyo, Japan) was used to measure bioelectrical impedance and obtain whole and segmental body composition data. This system uses three different frequency currents (5, 50, and 250 kHz) for high accuracy. An estimation formula for ASM in this model has been published, and a previous validation study demonstrated that body composition measured using this device was highly correlated with that measured using dual‐energy X‐ray absorptiometry measurements.^34^ Data were collected while participants stood on foot‐electrodes and held hand‐electrodes.

Thereafter, the skeletal muscle mass index (SMI, kg/m^2^) was calculated by dividing the ASM (kg) by the square of the height (m^2^). Body mass index (BMI, kg/m^2^) was calculated by the body weight (kg) by dividing the square of the height (m^2^).

Moreover, the PhA was calculated using the following formula: PhA (°) = −arctangent (Xc/R) * (180/π). Reactance and resistance values measured at 50 kHz current were used to calculate the PhA.^19^

#### Muscle quality

The EWGSOP2 sarcopenia guidelines define muscle quality as ‘referring both to micro‐ and macroscopic changes in muscle architecture and composition, and to muscle function delivered per unit of muscle mass’.^4^ In this study, muscle quality (kg/kg) was calculated by dividing handgrip strength (HGS) (kg) by upper limbs muscle mass (kg) according to previous reports,^17^, ^35^, ^36^ focusing on ‘muscle function delivered per unit of muscle mass’. HGS was measured using a Smedley spring‐type dynamometer (Grip‐D, Takei Scientific Instruments Co., Ltd., Niigata, Japan), while upper limb muscle mass was determined using BIA.

#### Sarcopenia definition

Sarcopenia was determined using HGS and the SMI based on algorithms proposed by EWGSOP2.^4^ Cut‐off values for HGS were set to <28 and <18 kg, while those for SMI were set to <7.0 and <5.7 kg/m^2^ for Asian male and female participants according to the guideline of Asian Working Group for Sarcopenia, respectively.^37^ Participants having a HGS and SMI below both criteria were determined to have sarcopenia.

### Statistical analysis

Data are expressed as mean ± standard deviation. All statistical analyses were performed using BellCurve for Excel Version 3.20 (Social Survey Research Information Co., Ltd., Tokyo, Japan), with a two‐sided *P* value of <0.05 indicating statistical significance.

The Mann–Whitney *U* test was used for assessing differences between young and elderly groups according to sex.

Single linear regression analysis was conducted to evaluate the association between PhA and each measured item. For multiple linear regression analysis of PhA, age, SMI, HGS, and muscle quality were selected as independent variables: Model 1 (independent variables: age and SMI), Model 2 (independent variables: age, SMI, and HGS), and Model 3 (independent variables: age, SMI, and muscle quality).

The Mann–Whitney *U* test was used to examine differences in PhA between the sarcopenia and non‐sarcopenia groups (young male, elderly male, young female, and elderly female participants). Moreover, receiver operating characteristic (ROC) curve and the area under the ROC curve (AUC) were used to determine the cut‐off values of PhA that indicated the presence of sarcopenia in each group. Cut‐off values were determined using the point on the ROC curve located closest to the (0, 1) point.

## Results

**Figure 1:**
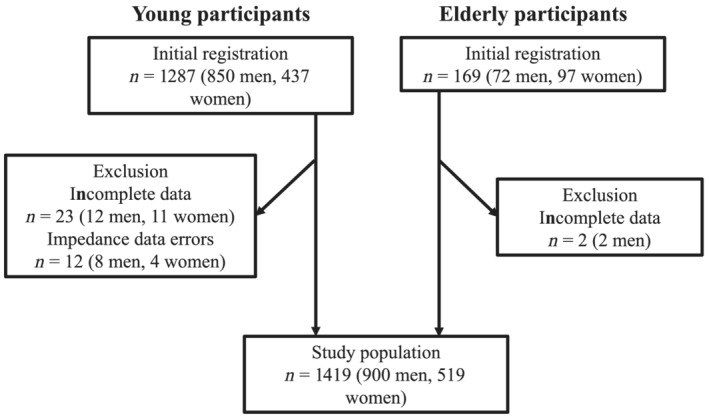
Study flow for participant registration.

Initially, 1456 individuals, comprising 1287 young (850 male and 437 female participants) and 169 elderly participants (72 male and 97 female participants) were registered in the present study. After conducting measurements, 23 young participants (12 males and 11 female participants) and 2 elderly male participants with incomplete data and 12 young participants (8 male and 4 female participants) with impedance data errors were excluded. Ultimately, 1419 participants composed of 830 young male, 70 elderly male, 422 young female, and 97 elderly female participants were included in the study population (*Figure* *1*).

Participant characteristics are summarized in *Table* *1*. Accordingly, young and elderly male participants had a mean PhA of 6.25 ± 0.50° and 5.26 ± 0.50° respectively, with the difference being significant (*P* < 0.001). Similarly, young female participants had a significantly higher the mean PhA compared with elderly ones (young: 5.37 ± 0.44°, elderly: 4.61 ± 0.43°; *P* < 0.001).

**Table 1:** Characteristics of participants

Male	All (*n* = 900)	Young (*n* = 830)	Elderly (*n* = 70)	*P* value
Age (year)	22.8 ± 15.1	18.5 ± 0.6	74.4 ± 5.5	<0.001
Height (m)	1.71 ± 0.06	1.71 ± 0.06	1.65 ± 0.06	<0.001
Body weight (kg)	62.4 ± 9.7	62.2 ± 9.8	63.8 ± 8.4	0.069
BMI (kg/m^2^)	21.4 ± 3.1	21.2 ± 3.0	23.4 ± 2.6	<0.001
Total fat mass (kg)	11.5 ± 5.4	11.2 ± 5.3	14.2 ± 5.2	<0.001
Body fat percentage (%)	17.7 ± 5.7	17.4 ± 5.6	21.7 ± 5.8	<0.001
Total muscle mass (kg)	48.3 ± 5.1	48.4 ± 5.2	47.0 ± 4.4	0.037
ASM (kg)	23.1 ± 2.9	23.3 ± 2.8	21.6 ± 3.0	<0.001
SMI (kg/m^2^)	7.9 ± 0.7	7.9 ± 0.7	7.9 ± 0.8	0.893
Total bone mass (kg)	2.7 ± 0.3	2.7 ± 0.3	2.6 ± 0.2	0.039
Body water content (kg)	34.6 ± 4.1	34.5 ± 4.1	35.9 ± 4.1	0.006
HGS (kg)	38.4 ± 6.1	38.6 ± 6.0	35.7 ± 6.7	<0.001
Muscle quality (kg/kg)	8.3 ± 1.1	8.4 ± 1.1	7.5 ± 1.1	<0.001
Phase angle (°)	6.17 ± 0.57	6.25 ± 0.50	5.26 ± 0.50	<0.001

Female	All (*n* = 519)	Young (*n* = 422)	Elderly (*n* = 97)	*P* value
Age (year)	28.5 ± 21.6	18.3 ± 0.5	73.1 ± 6.4	<0.001
Height (m)	1.57 ± 0.06	1.58 ± 0.05	1.52 ± 0.06	<0.001
Body weight (kg)	51.5 ± 7.1	51.5 ± 6.8	51.4 ± 8.2	0.499
BMI (kg/m^2^)	20.8 ± 2.6	20.5 ± 2.3	22.2 ± 3.1	<0.001
Total fat mass (kg)	15.1 ± 4.9	14.9 ± 4.5	15.9 ± 6.4	0.162
Body fat percentage (%)	28.8 ± 5.5	28.5 ± 4.8	30.0 ± 7.6	0.007
Total muscle mass (kg)	34.3 ± 3.0	34.5 ± 3.0	33.6 ± 3.1	0.013
ASM (kg)	15.9 ± 1.8	16.1 ± 1.7	15.1 ± 2.1	<0.001
SMI (kg/m^2^)	6.4 ± 0.5	6.4 ± 0.4	6.5 ± 0.5	0.087
Total bone mass (kg)	2.0 ± 0.3	2.04 ± 0.28	1.95 ± 0.29	0.012
Body water content (kg)	25.8 ± 2.6	25.6 ± 2.5	26.4 ± 2.9	0.025
HGS (kg)	25.1 ± 4.6	25.8 ± 4.3	22.0 ± 4.9	<0.001
Muscle quality (kg/kg)	8.5 ± 1.4	8.8 ± 1.2	7.2 ± 1.3	<0.001
Phase angle (°)	5.22 ± 0.53	5.37 ± 0.44	4.61 ± 0.43	<0.001

ASM, appendicular skeletal muscle; BMI, body mass index; HGS, handgrip strength; SMI, skeletal mass index.

Data are expressed as mean ± standard deviation. Significant differences between young and elderly subjects were determined using the Mann–Whitney *U* test.

*Table* *2* details the results of single linear regression analysis on the relationship between PhA and measured items. In both male and female participants, a negative correlation was observed between PhA and age (male: *r* = −0.48, *P* < 0.001; female: *r* = −0.57, *P* < 0.001). PhA was positively correlated with both the SMI and HGS, which were diagnostic indicators for sarcopenia [SMI (male: *r* = 0.59, *P* < 0.001; female: *r* = 0.50, *P* < 0.001) and HGS (male: *r* = 0.40, *P* < 0.001; female: *r* = 0.47, *P* < 0.001)]. Furthermore, PhA was positively correlated with muscle quality (male: *r* = 0.09, *P* = 0.006; female: *r* = 0.36, *P* < 0.001).

**Table 2:** Single linear regression analysis on the relationship between phase angle and measured items

Variable	Male		Female	
	*r*	*P* value	*r*	*P* value
Age (year)	−0.48	<0.001	−0.57	<0.001
Height (m)	−0.04	0.250	0.15	<0.001
Body weight (kg)	0.28	<0.001	0.13	0.002
BMI (kg/m^2^)	0.32	<0.001	0.05	0.229
Total fat mass (kg)	0.08	0.014	−0.03	0.564
Body fat percentage (%)	−0.02	0.540	−0.12	0.004
Total muscle mass (kg)	0.35	<0.001	0.33	<0.001
ASM (kg)	0.41	<0.001	0.40	<0.001
SMI (kg/m^2^)	0.59	<0.001	0.50	<0.001
Total bone mass (kg)	0.42	<0.001	0.33	<0.001
Body water content (kg)	0.43	<0.001	0.25	<0.001
HGS (kg)	0.40	<0.001	0.47	<0.001
Muscle quality (kg/kg)	0.09	0.006	0.36	<0.001

ASM, appendicular skeletal muscle; BMI, body mass index; HGS, handgrip strength; SMI, skeletal mass index.

Results are expressed as correlation coefficients (*r*).

The results of multiple linear regression analysis are provided in *Table* *3*. First, age and SMI, which were strongly correlated with PhA, were selected as independent variables. Thereafter, HGS (a diagnostic indicator for sarcopenia) and muscle quality were also selected as independent variables. In this study, we particularly focused on the relationship between muscle quality and PhA. Model 1 showed that both of age and SMI were significantly associated with PhA. Model 2 showed that only HGS was not significantly associated with PhA, although age and SMI were significantly associated. Model 3 showed that all of age, SMI, and muscle quality significantly associated with PhA in both male and female participants. Age was negatively associated, and SMI and muscle quality were positively associated, respectively [male (age: standardized regression coefficient (*β*) = −0.43, *P* < 0.001, SMI: *β* = 0.61, *P* < 0.001, muscle quality: *β* = 0.13, *P* < 0.001); female (age: *β* = −0.56, *P* < 0.001, SMI: *β* = 0.52, *P* < 0.001, muscle quality: *β* = 0.09, *P* = 0.007)].

**Table 3:** Multiple linear regression analysis on phase angle

Male	Model 1				Model 2				Model 3			
	95% CI		*β*	*P* value	95% CI		*β*	*P* value	95% CI		*β*	*P* value
	Lower	Upper			Lower	Upper			Lower	Upper		
Age (year)	−0.019	−0.016	−0.46	<0.001	−0.019	−0.016	−0.46	<0.001	−0.018	−0.015	−0.43	<0.001
SMI (kg/m^2^)	0.419	0.486	0.58	<0.001	0.397	0.477	0.56	<0.001	0.442	0.511	0.61	<0.001
HGS (kg)	—	—	—	—	−0.001	0.008	0.04	0.162	—	—	—	—
Muscle quality (kg/kg)	—	—	—	—	—	—	—	—	0.047	0.094	0.13	<0.001

Female												
Age (year)	−0.016	−0.013	−0.60	<0.001	−0.016	−0.013	−0.60	<0.001	−0.015	−0.012	−0.56	<0.001
SMI (kg/m^2^)	0.547	0.672	0.53	<0.001	0.535	0.687	0.53	<0.001	0.542	0.667	0.52	<0.001
HGS (kg)	—	—	—	—	−0.008	0.008	0.00	0.940	—	—	—	—
Muscle quality (kg/kg)	—	—	—	—	—	—	—	—	0.009	0.057	0.09	0.007

*β*, standardized regression coefficient; CI, confidence interval; HGS, handgrip strength; SMI, skeletal mass index.

Model 1: age and SMI; Model 2: age, SMI, and HGS; Model 3: age, SMI, and muscle quality.

*Table* *4* shows comparisons of the PhA values between the sarcopenia and non‐sarcopenia groups. Sarcopenia was diagnosed in eight young male (1.0%), two elderly male (2.9%), five young female (1.2%), and five elderly female (5.2%) participants. Young male, young female, and elderly female participants in the sarcopenia group had significantly lower mean PhA than did those in the non‐sarcopenia group. Although no significant difference was observed among elderly male participants, the same tendency was noted.

**Table 4:** Comparisons of PhA values between the non‐sarcopenia and sarcopenia groups

Sex	Age	Non‐sarcopenia	*N*	Sarcopenia	*N*	*P* value
Male	Young	6.25 ± 0.50	822	5.51 ± 0.41	8	<0.001
	Elderly	5.28 ± 0.48	68	4.48 ± 0.78	2	N.A.
Female	Young	5.37 ± 0.44	417	4.88 ± 0.16	5	0.005
	Elderly	4.63 ± 0.42	92	4.14 ± 0.29	5	0.009

N.A., not analysed; PhA, phase angle.

Data are expressed as mean ± standard deviation. Significant differences between the non‐sarcopenia and sarcopenia groups were determined using the Mann–Whitney *U* test.

**Figure 2:**
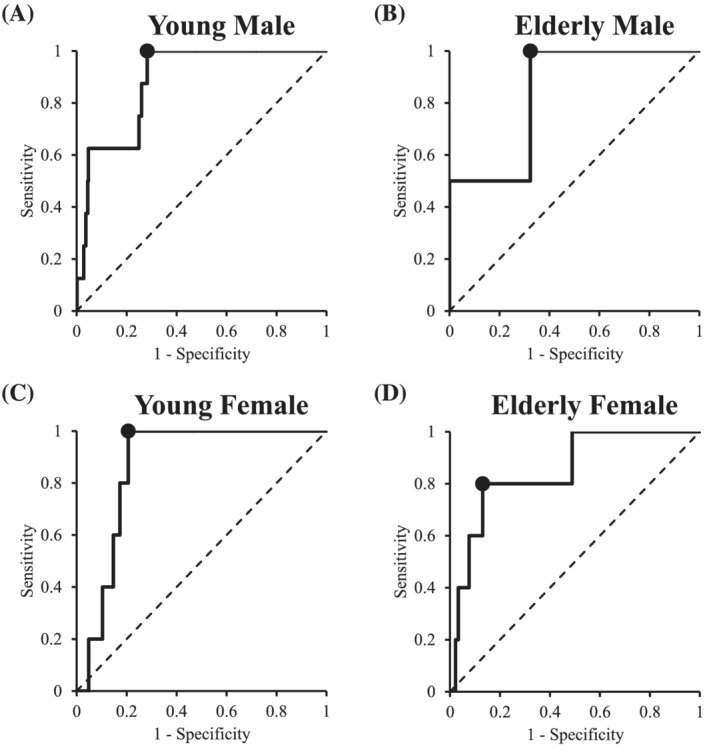
Receiver operating characteristic (ROC) curve and area under the ROC curve (AUC) of PhA to detect sarcopenia in young male *(A)*, elderly male *(B)*, young female *(C)*, and elderly female *(D)* participants.

Finally, results of the ROC analyses are presented in *Figure* *2* and *Table* *5*. The results showed moderate predictive accuracy of PhA for sarcopenia in young male (AUC = 0.882, 95% CI = 0.796–0.967), elderly male (AUC = 0.838, 95% CI = 0.516–1.160), young female (AUC = 0.865, 95% CI = 0.804–0.926), and elderly female (AUC = 0.850, 95% CI = 0.674–1.026) participants, respectively. The cut‐off values of PhA to discriminate sarcopenia from non‐sarcopenia were 5.95° in young male participants, 5.04° in elderly male participants, 5.02° in young female participants, and 4.20° in elderly female participants, respectively.

**Table 5:** Predictive ability of PhA and cut‐off values for sarcopenia

Sex	Age	AUC	95% CI	*P* value	Cut‐off (°)	Sensitivity (%)	Specificity (%)
Male	Young	0.882	0.796–0.967	<0.001	5.95	100.0	71.8
	Elderly	0.838	0.516–1.160	0.039	5.04	100.0	67.6
Female	Young	0.865	0.804–0.926	<0.001	5.02	100.0	79.4
	Elderly	0.850	0.674–1.026	<0.001	4.20	80.0	87.0

AUC, area under the receiver operating characteristic curve; CI, confidence interval; PhA, phase angle.

## Discussion

To the best of our knowledge, this is the first study demonstrating that PhA can be an independent useful indicator of muscle quality defined as HGS divided by upper limbs muscle mass. This study showed that PhA was independently associated with age, SMI, and muscle quality. Our results also showed that the sarcopenia group had a significantly lower PhA than the non‐sarcopenia group. Moreover, ROC analysis revealed that PhA exhibited good accuracy in predicting sarcopenia while indicating the best cut‐off values of PhA.

Indeed, studies have shown that PhA decreased with age^23^, ^24^, ^25^ was lower in women than in men^23^, ^24^, ^25^ and was proportional to BMI.^23^, ^24^, ^25^ Moreover, other reports have shown that the PhA was positively correlated with muscle mass, HGS,^38^ lower limb muscle strength,^33^ and physical function assessed using the speed at which individuals walked and rose from the sitting position.^39^ The present study found that PhA was lower in women than in men, was negatively correlated with age, and was positively correlation with BMI, muscle mass, and HGS. While these results were consistent with those presented in previous reports, our finding showed that SMI had the strongest correlation with PhA among the body composition and physical function parameters (*Table* *2*).

One study showed that PhA was moderately positively correlation with muscle quality defined as HGS divided by upper limbs muscle mass among elderly women.^39^ Similarly, the present study found a moderately positive correlation between PhA and muscle quality among female participants (*r* = 0.36, *P* < 0.001), although a very weak positive correlation had been noted among male participants (*r* = 0.09, *P* = 0.006). However, multiple linear regression analysis conducted herein revealed that PhA was significantly associated with age, SMI, and muscle quality in both male (*β* = 0.13, *P* < 0.001) and female (*β* = 0.09, *P* = 0.007) participants. One interesting finding obtained herein was that muscle quantity and quality were each independently positively correlated with PhA, suggesting that higher PhA indicated higher muscle quantity and quality.

Evidence has shown that the PhA reflects cell membrane structure, cell mass, cellular integrity, and cell function, with higher PhA levels indicating better parameters.^20^, ^22^, ^40^ Although the relationship between the PhA and muscle parameters has yet to be fully elucidated, the PhA values were independently associated with muscle quality defined as HGS divided by upper limb muscle mass. The decrease in PhA has been attributed to a reduction in reactance due to muscle mass loss and/or increased resistance caused by increased fat mass.^22^ Increased intramuscular fat and fibrous tissue has been considered to decrease muscle quality.^10^ As such, changes in muscle mass and quality have been considered to be independently associated with the PhA. In line with this, sarcopenia guidelines have indicated the need for examining not only muscle quantity but also muscle quality.^4^ Considering that PhA measurements can simultaneously determine both muscle quality and quantity, they can perhaps be utilized to satisfy requirements established by the guidelines.

Our findings showed that elderly female participants in the sarcopenia group had lower PhA values than those in the non‐sarcopenia group. Although no significant difference had been observed in elderly male participants, the same tendency had been noted. This could have been attributed to the fact that only two participants were diagnosed with sarcopenia. These results were consistent with those presented in a previous report, wherein elderly individuals with sarcopenia had lower PhA values than those without sarcopenia.^41^ The present study also showed that young participants in the sarcopenia group had lower PhA values that those in the non‐sarcopenia group.

Moreover, ROC analyses revealed that all young male, young female, elderly male, and elderly female participants had an AUC exceeding 0.8, indicating that PhA had good accuracy in predicting sarcopenia, with cut‐off values of 5.95°, 5.04°, 5.02°, and 4.20°, respectively.

Interestingly, elderly individuals included herein had higher cut‐off PhA values compared with those included in previous studies,^41^, ^42^ perhaps because our elderly participants were younger. Given that the current study and previous studies^23^, ^24^, ^25^ have shown that PhA decreases with age, cut‐off values may be considered to differ depending on the age of the target population. Therefore, using PhA for sarcopenia diagnosis requires establishing cut‐off values for not only sex but also 5 or 10 year age groups.

Although differences in PhA had been observed and factors associated with PhA had been identified, the current study could not determine how the decrease in PhA over time adversely affects health given the cross‐sectional design of the current study. Studies have suggested that PhA may increase following short‐term resistance training.^43^ Therefore, to evaluate the effects of changes in PhA over time on physical health, further longitudinal studies are necessary.

The EWGSOP2 guideline has defined ‘muscle quality’ as that ‘referring both to micro‐ and macroscopic changes in muscle architecture and composition, and to muscle function delivered per unit of muscle mass’.^4^ However, these definitions are not identical. Although increased levels of intramuscular fat have been reported to lead muscle dysfunction, such as decreased muscle strength,^44^, ^45^ their minute association remains to be elucidated in the future. Investigating fat infiltration into muscles using computed tomography and magnetic resonance imaging, as well as muscle echo intensity using ultrasonography images, in conjunction with PhA measurements, thereby evaluating the relationship between PhA and changes in muscle architecture and composition, would certainly improve the utility of PhA as a marker of muscle quality.

In addition, subtle differences in PhA values could exist due to differences in BIA measuring equipment. Given that PhA is calculated directly from the electrical opposition value of the body without using an estimation formula, unlike body composition data (e.g. lean body mass and body fat percentage), differences between device manufacturers have been considered small. However, no studies have examined differences among measuring instruments. For the effective use of PhA in diagnosing sarcopenia, differences in equipment need to be considered.

There were several limitations that warrant mention. First, our study was cross‐sectional in design. Thus, it could only examine associations between the PhA and muscle quality and muscle‐related parameters. Larger cohort and prospective studies are necessary to determine any causal relationships in the future. Second, there was the discrepancy in the sample sizes of young and elderly participants (young vs. elderly: 1252 participants vs. 167 participants). If possible, it would be desirable to compare and analyse the elderly participants with the comparable sample size as the young participants. However, it was not possible to control the number of participants who received health check‐ups. Then, we examined only the elderly participants in Seika town. Because of the small number of participants analysed, the multiple linear regression analysis on PhA was performed for male and female participants combined; however, we found similar results that age, SMI, and muscle quality were independently associated with PhA (data not shown). As we diagnosed sarcopenia according to the criteria of AWGS2019, we believe that the conclusions obtained will remain the same. In the future, further validation by large‐scale participants will be necessary. Third, it is possible that there was a bias in the participants of this study: the elderly participants were those who underwent specific health check‐ups in Seika town and were relatively healthy. Therefore, the prevalence of sarcopenia in the elderly participants was lower (elderly male participants, 2.9%; elderly female participants 5.2%) than previously reported. However, we believe that the conclusions obtained will remain the same, as we diagnosed sarcopenia according to the criteria of AWGS2019. In the future, further validation by large‐scale participants will be necessary. Fourth, sarcopenia in this study was diagnosed by low SMI and low HGS according to the guidelines of EWGSOP2. Decline in physical function is also an important aspect of sarcopenia. The association between PhA and physical function is a subject for further investigation. Fifth, we did not directly measure muscle quality, such as with imaging analysis using ultrasonography or computed tomography. A detailed evaluation of muscle quality might further clarify the harmful effects of diabetes on physical performance.

In conclusion, the current study revealed that PhA was associated with age, SMI, and muscle quality and that higher PhA reflected higher muscle quality. Moreover, this study demonstrated that PhA had good accuracy for detecting sarcopenia. Cut‐off PhA values for predicting sarcopenia were 5.95°, 5.04°, 5.02°, and 4.20° in young male, elderly male, young female, and elderly female participants, respectively. Taken together, these novel findings indicate that PhA can be a useful index for easily measuring muscle quality, which has been desired when diagnosing sarcopenia.

## Conflict of interest

All authors of this manuscript declare no conflicts of interest.

## References

[1] Santilli V , Bernetti A , Mangone M , Paoloni M . Clinical definition of sarcopenia. Clin Cases Miner Bone Metab 2014;11:177–180. 25568649 PMC4269139

[2] Shafiee G , Keshtkar A , Soltani A , Ahadi Z , Larijani B , Heshmat R . Prevalence of sarcopenia in the world: a systematic review and meta‐ analysis of general population studies. J Diabetes Metab Disord 2017;16:21. 28523252 10.1186/s40200-017-0302-x PMC5434551

[3] Cruz‐Jentoft AJ , Baeyens JP , Bauer JM , Boirie Y , Cederholm T , Landi F , et al. Sarcopenia: European consensus on definition and diagnosis: report of the European Working Group on Sarcopenia in Older People. Age Ageing 2010;39:412–423. 20392703 10.1093/ageing/afq034 PMC2886201

[4] Cruz‐Jentoft AJ , Bahat G , Bauer J , Boirie Y , Bruyere O , Cederholm T , et al. Sarcopenia: revised European consensus on definition and diagnosis. Age Ageing 2019;48:16–31. 30312372 10.1093/ageing/afy169 PMC6322506

[5] Freilich RJ , Kirsner RL , Byrne E . Isometric strength and thickness relationships in human quadriceps muscle. Neuromuscul Disord 1995;5:415–422. 7496175 10.1016/0960-8966(94)00078-n

[6] Singer KP , Breidahl P . The use of computed tomography in assessing muscle cross‐sectional area, and the relationship between cross‐sectional area and strength. Aust J Physiother 1987;33:75–82. 25025589 10.1016/S0004-9514(14)60585-7

[7] Ferrucci L , de Cabo R , Knuth ND , Studenski S . Of Greek heroes, wiggling worms, mighty mice, and old body builders. J Gerontol A Biol Sci Med Sci 2012;67:13–16. 22113943 10.1093/gerona/glr046 PMC3260484

[8] Cruz‐Jentoft AJ , Sayer AA . Sarcopenia. Lancet 2019;393:2636–2646. 31171417 10.1016/S0140-6736(19)31138-9

[9] Berger MJ , Doherty TJ . Sarcopenia: prevalence, mechanisms, and functional consequences. Interdiscip Top Gerontol 2010;37:94–114. 20703058 10.1159/000319997

[10] McGregor RA , Cameron‐Smith D , Poppitt SD . It is not just muscle mass: a review of muscle quality, composition and metabolism during ageing as determinants of muscle function and mobility in later life. Longev Healthspan 2014;3:9. 25520782 10.1186/2046-2395-3-9 PMC4268803

[11] Hamaguchi Y , Kaido T , Okumura S , Kobayashi A , Shirai H , Yagi S , et al. Impact of skeletal muscle mass index, intramuscular adipose tissue content, and visceral to subcutaneous adipose tissue area ratio on early mortality of living donor liver transplantation. Transplantation 2017;101:565–574. 27926595 10.1097/TP.0000000000001587

[12] Karampinos DC , Baum T , Nardo L , Alizai H , Yu H , Carballido‐Gamio J , et al. Characterization of the regional distribution of skeletal muscle adipose tissue in type 2 diabetes using chemical shift‐based water/fat separation. J Magn Reson Imaging 2012;35:899–907. 22127958 10.1002/jmri.23512 PMC3292710

[13] Goodpaster BH , Kelley DE , Thaete FL , He J , Ross R . Skeletal muscle attenuation determined by computed tomography is associated with skeletal muscle lipid content. J Appl Physiol (1985) 2000;89:104–110. 10904041 10.1152/jappl.2000.89.1.104

[14] Fukumoto Y , Ikezoe T , Yamada Y , Tsukagoshi R , Nakamura M , Mori N , et al. Skeletal muscle quality assessed from echo intensity is associated with muscle strength of middle‐aged and elderly persons. Eur J Appl Physiol 2012;112:1519–1525. 21847576 10.1007/s00421-011-2099-5

[15] Lynch NA , Metter EJ , Lindle RS , Fozard JL , Tobin JD , Roy TA , et al. Muscle quality. I. Age‐associated differences between arm and leg muscle groups. J Appl Physiol (1985) 1999;86:188–194. 9887130 10.1152/jappl.1999.86.1.188

[16] Tracy BL , Ivey FM , Hurlbut D , Martel GF , Lemmer JT , Siegel EL , et al. Muscle quality. II. Effects of strength training in 65‐ to 75‐yr‐old men and women. J Appl Physiol (1985) 1999;86:195–201. 9887131 10.1152/jappl.1999.86.1.195

[17] Murai J , Nishizawa H , Otsuka A , Fukuda S , Tanaka Y , Nagao H , et al. Low muscle quality in Japanese type 2 diabetic patients with visceral fat accumulation. Cardiovasc Diabetol 2018;17:112. 30077183 10.1186/s12933-018-0755-3 PMC6076400

[18] Kyle UG , Bosaeus I , De Lorenzo AD , Deurenberg P , Elia M , Manuel Gomez J , et al. Bioelectrical impedance analysis‐part II: utilization in clinical practice. Clin Nutr 2004;23:1430–1453. 15556267 10.1016/j.clnu.2004.09.012

[19] Kyle UG , Bosaeus I , De Lorenzo AD , Deurenberg P , Elia M , Gomez JM , et al. Bioelectrical impedance analysis—part I: review of principles and methods. Clin Nutr 2004;23:1226–1243. 15380917 10.1016/j.clnu.2004.06.004

[20] Lukaski HC . Evolution of bioimpedance: a circuitous journey from estimation of physiological function to assessment of body composition and a return to clinical research. Eur J Clin Nutr 2013;67:S2–S9. 23299867 10.1038/ejcn.2012.149

[21] Sardinha LB . Physiology of exercise and phase angle: another look at BIA. Eur J Clin Nutr 2018;72:1323–1327. 30185857 10.1038/s41430-018-0215-x

[22] Norman K , Stobaus N , Pirlich M , Bosy‐Westphal A . Bioelectrical phase angle and impedance vector analysis—clinical relevance and applicability of impedance parameters. Clin Nutr 2012;31:854–861. 22698802 10.1016/j.clnu.2012.05.008

[23] Barbosa‐Silva MC , Barros AJ , Wang J , Heymsfield SB , Pierson RN Jr . Bioelectrical impedance analysis: population reference values for phase angle by age and sex. Am J Clin Nutr 2005;82:49–52. 16002799 10.1093/ajcn.82.1.49

[24] Bosy‐Westphal A , Danielzik S , Dorhofer RP , Later W , Wiese S , Muller MJ . Phase angle from bioelectrical impedance analysis: population reference values by age, sex, and body mass index. JPEN J Parenter Enteral Nutr 2006;30:309–316. 16804128 10.1177/0148607106030004309

[25] Dittmar M . Reliability and variability of bioimpedance measures in normal adults: effects of age, gender, and body mass. Am J Phys Anthropol 2003;122:361–370. 14614757 10.1002/ajpa.10301

[26] Slee A , Birc D , Stokoe D . Bioelectrical impedance vector analysis, phase‐angle assessment and relationship with malnutrition risk in a cohort of frail older hospital patients in the United Kingdom. Nutrition 2015;31:132–137. 25466657 10.1016/j.nut.2014.06.002

[27] Ott M , Fischer H , Polat H , Helm EB , Frenz M , Caspary WF , et al. Bioelectrical impedance analysis as a predictor of survival in patients with human immunodeficiency virus infection. J Acquir Immune Defic Syndr Hum Retrovirol 1995;9:20–25. 7712230

[28] Schwenk A , Beisenherz A , Romer K , Kremer G , Salzberger B , Elia M . Phase angle from bioelectrical impedance analysis remains an independent predictive marker in HIV‐infected patients in the era of highly active antiretroviral treatment. Am J Clin Nutr 2000;72:496–501. 10919947 10.1093/ajcn/72.2.496

[29] do Amaral Paes TC , de Oliveira KCC , de Carvalho Padilha P , Peres WAF . Phase angle assessment in critically ill cancer patients: relationship with the nutritional status, prognostic factors and death. J Crit Care 2018;44:430–435. 29353120 10.1016/j.jcrc.2018.01.006

[30] Toso S , Piccoli A , Gusella M , Menon D , Bononi A , Crepaldi G , et al. Altered tissue electric properties in lung cancer patients as detected by bioelectric impedance vector analysis. Nutrition 2000;16:120–124. 10696635 10.1016/s0899-9007(99)00230-0

[31] Toso S , Piccoli A , Gusella M , Menon D , Crepaldi G , Bononi A , et al. Bioimpedance vector pattern in cancer patients without disease versus locally advanced or disseminated disease. Nutrition 2003;19:510–514. 12781850 10.1016/s0899-9007(02)01084-5

[32] Maggiore Q , Nigrelli S , Ciccarelli C , Grimaldi C , Rossi GA , Michelassi C . Nutritional and prognostic correlates of bioimpedance indexes in hemodialysis patients. Kidney Int 1996;50:2103–2108. 8943496 10.1038/ki.1996.535

[33] Yamada Y , Buehring B , Krueger D , Anderson RM , Schoeller DA , Binkley N . Electrical properties assessed by bioelectrical impedance spectroscopy as biomarkers of age‐related loss of skeletal muscle quantity and quality. J Gerontol A Biol Sci Med Sci 2017;72:1180–1186. 28814064 10.1093/gerona/glw225 PMC5861891

[34] Yamada Y , Nishizawa M , Uchiyama T , Kasahara Y , Shindo M , Miyachi M , et al. Developing and validating an age‐independent equation using multi‐frequency bioelectrical impedance analysis for estimation of appendicular skeletal muscle mass and establishing a cutoff for sarcopenia. Int J Environ Res Public Health 2017;14. 10.3390/ijerph14070809 PMC5551247 28753945

[35] Cooper R , Hardy R , Bann D , Aihie Sayer A , Ward KA , Adams JE , et al. Body mass index from age 15 years onwards and muscle mass, strength, and quality in early old age: findings from the MRC National Survey of Health and Development. J Gerontol A Biol Sci Med Sci 2014;69:1253–1259. 24682351 10.1093/gerona/glu039 PMC4158414

[36] Lees MJ , Wilson OJ , Hind K , Ispoglou T . Muscle quality as a complementary prognostic tool in conjunction with sarcopenia assessment in younger and older individuals. Eur J Appl Physiol 2019;119:1171–1181. 30806780 10.1007/s00421-019-04107-8 PMC6469623

[37] Chen LK , Woo J , Assantachai P , Auyeung TW , Chou MY , Iijima K , et al. Asian working group for sarcopenia: 2019 consensus update on sarcopenia diagnosis and treatment. J Am Med Dir Assoc 2020;21:300–307, e302. 32033882 10.1016/j.jamda.2019.12.012

[38] Basile C , Della‐Morte D , Cacciatore F , Gargiulo G , Galizia G , Roselli M , et al. Phase angle as bioelectrical marker to identify elderly patients at risk of sarcopenia. Exp Gerontol 2014;58:43–46. 25034911 10.1016/j.exger.2014.07.009

[39] Tomeleri CM , Cavalcante EF , Antunes M , Nabuco HCG , de Souza MF , Teixeira DC , et al. Phase angle is moderately associated with muscle quality and functional capacity, independent of age and body composition in older women. J Geriatr Phys Ther 2019;42:281–286. 29210931 10.1519/JPT.0000000000000161

[40] De Lorenzo A , Andreoli A , Matthie J , Withers P . Predicting body cell mass with bioimpedance by using theoretical methods: a technological review. J Appl Physiol (1985) 1997;82:1542–1558. 9134904 10.1152/jappl.1997.82.5.1542

[41] Yamada M , Kimura Y , Ishiyama D , Nishio N , Otobe Y , Tanaka T , et al. Phase angle is a useful indicator for muscle function in older adults. J Nutr Health Aging 2019;23:251–255.3082051310.1007/s12603-018-1151-0

[42] Kilic MK , Kizilarslanoglu MC , Arik G , Bolayir B , Kara O , Dogan Varan H , et al. Association of bioelectrical impedance analysis‐derived phase angle and sarcopenia in older adults. Nutr Clin Pract 2017;32:103–109. 27590205 10.1177/0884533616664503

[43] Cunha PM , Tomeleri CM , Nascimento MAD , Nunes JP , Antunes M , Nabuco HCG , et al. Improvement of cellular health indicators and muscle quality in older women with different resistance training volumes. J Sports Sci 2018;36:2843–2848. 29790430 10.1080/02640414.2018.1479103

[44] Addison O , Marcus RL , Lastayo PC , Ryan AS . Intermuscular fat: a review of the consequences and causes. Int J Endocrinol 2014;2014:309570. 24527032 10.1155/2014/309570 PMC3910392

[45] Correa‐de‐Araujo R , Addison O , Miljkovic I , Goodpaster BH , Bergman BC , Clark RV , et al. Myosteatosis in the context of skeletal muscle function deficit: an interdisciplinary workshop at the National Institute on Aging. Front Physiol 2020;11:963. 32903666 10.3389/fphys.2020.00963 PMC7438777

[46] von Haehling S , Morley JE , Coats AJS , Anker SD . Ethical guidelines for publishing in the Journal of Cachexia, Sarcopenia and Muscle: update. J Cachexia Sarcopenia Muscle 2019;10:1143–1145. 31661195 10.1002/jcsm.12501 PMC6818444

